# Efficacy and Safety of Traditional Chinese Medicine in the Treatment of Immune Infertility Based on the Theory of “Kidney Deficiency and Blood Stasis”: A Systematic Review and Meta-Analysis

**DOI:** 10.1155/2021/9947348

**Published:** 2021-05-15

**Authors:** Yi-ling Bai, Yun-hui Chen, Cui Jiang, Jun-hui Qian, Ling-ling Han, Hai-zhen Lu, Hao-zhong Wang, Yi-rong Sun

**Affiliations:** ^1^College of Basic Medicine, Chengdu University of Traditional Chinese Medicine, Chengdu, Sichuan, China; ^2^Hospital of Chengdu University of Traditional Chinese Medicine, No. 39 Shi-er-qiao Road, Chengdu, Sichuan 610072, China; ^3^Guangzhou Institutes of Biomedicine and Health, Chinese Academy of Sciences, Guangzhou, China

## Abstract

**Objective:**

This study aims to evaluate the efficacy and safety of traditional Chinese medicine (TCM) therapy of tonifying kidney and activating blood circulation (TKABC) based on the theory of “kidney deficiency and blood stasis” for the treatment of immune infertility.

**Methods:**

Six electronic databases, including the Cochrane Library, PubMed, EMBASE, the China National Knowledge Infrastructure, Wanfang Data, and VIP information database, were searched from inception to January 2021 to identify eligible studies of randomized controlled trials (RCTs). The primary outcome measurements were the total effective rate and pregnancy rate, and the secondary outcome measurements included the negative conversion rate of serum antibodies and the incidence of adverse effects. The quantitative synthesis was performed using the Review Manager 5.3 software. The chi-square statistic and *I*^2^ statistic were employed to investigate statistical heterogeneity. The fixed-effects model was used for a low heterogeneity (*I*^2^ < 50%), and the random-effects model was applied if heterogeneity was moderate (50% < *I*^2^ < 75%). Funnel plots were used to evaluate potential reporting bias when more than ten eligible studies were included.

**Results:**

Thirteen RCTs involving 1298 patients with immune infertility of kidney deficiency and blood stasis were included. Compared with conventional group, TCM TKABC therapy showed a significant improvement on the total effective rate (RR: 1.38; 95% CI: 1.30,1.47; and *I*^2^ = 0%), pregnancy rate (RR: 2.04; 95% CI: 1.73, 2.40; and *I*^2^ = 30%), negative conversion rates of AsAb (RR: 1.42; 95% CI: 1.12,1.79; and *I*^2^ = 62%), AEmAb rates (RR: 1.21; 95% CI: 1.04,1.41; and I^2^ = 0%), and AhCGAb with less adverse effects (RR: 0.24; 95% CI: 1.73, 2.40; and *I*^2^ = 55%). However, the negative conversion rate of AoAb and ACAb showed no significant statistical difference.

**Conclusions:**

Our review suggests that TCM TKABC therapy based on the theory of kidney deficiency and blood stasis appears to be an effective and safe approach for patients with immune infertility. However, the methodological quality of included RCTs was unsatisfactory, and it is necessary to verify its effectiveness with more well-designed and high-quality multicenter RCTs.

## 1. Introduction

Immune infertility is defined as the presence, in one or both partners, of an antisperm immune reaction capable of impairing fertility variables [1]. It has become a serious health issue as approximately 10 to 20 percent of the sterility cases are immunological [2]. Although the definitive cause of immune infertility remains ambiguous, the presence of antireproductive antibodies in serum has been elucidated as one of the major causes of immune infertility. It has been reported that the presence of such antibodies as antisperm (AsAb), antiendometrium (AEmAb), antiovary (AoAb), antihuman chorionic gonadotropin (AhCGAb), antizona pellucida (AZPAb), antitrophoblast (ATB), and anticardiolipin (ACA) may affect fertilization and implantation process, resulting in infertility [3]. The primary conventional treatment choices include immunosuppressive drugs, anticoagulants, intrauterine insemination, and *in vitro* fertilization. However, long-term usage of immunosuppressive therapy may cause side effects, and assisted reproduction treatment is expensive with a low success rate [3, 4]. Hence, in recent years, the interest in complementary and alternative medicine has increased.

Traditional Chinese medicine (TCM) has been commonly used to treat infertility in Asian countries. TCM is featured by the concept of holism and treatment based on syndrome differentiation. From the perspective of TCM, immune infertility is often attributable to kidney deficiency and blood stasis [5]. Previous studies reported that TCM therapy of tonifying kidney and activating blood circulation (Bushen Huoxue, TKABC) is essential for treating this illness [5, 6]. A large number of studies have reported that TKABC may remarkably reduce serum levels of such antibodies as AsAb, eliminate testicular immunological complexes, regulate the ratio of CD_4_/CD_8_ T cells, and eliminate inflammatory cytokines to cure immune-induced infertility [7–10]. In recent years, a growing body of random controlled trials (RCTs) has been conducted to assess the effectiveness and safety of TKABC therapy for the treatment of immune infertility, and the results have suggested it might be an effective and safe therapeutic approach. However, currently no systematic review and meta-analysis have been reported for this specific ailment. Thus, we performed this study to evaluate the efficacy and safety of TCM TKABC therapy based on the theory of “kidney deficiency and blood stasis” for the treatment of immune infertility. Hopefully, the findings of this review may provide helpful evidence for the decision-making process of the patients, physicians, and investigators concerned.

## 2. Methods

This meta-analysis was conducted using Review Manager following the Cochrane Handbook for Systematic Reviews of Interventions (version 5.3.3) and the Preferred Reporting Items for Systematic Reviews and Meta-Analyses guidelines. The protocol of this review was registered in INPLASY (INPLASY202110098).

### 2.1. Search Strategy

Six electronic databases, including China National Knowledge Infrastructure (CNKI), Wanfang Data, Chinese Scientific Journals Database (VIP), PubMed, EMBASE, and Cochrane Library were searched from inception to January 2021 for identifying eligible studies. No restriction on language or publication status was imposed. The following terms were used in a combination for the electronic search: immune infertility, immunological infertility, infertility, traditional Chinese medicine, complementary and alternative medicine, Chinese medicine, herbal medicine, prescription, formula, kidney deficiency, blood stagnation, blood stasis, supplementing kidney, tonifying kidney, activating blood circulation, randomized control, randomization, randomized clinical trials, RCT, and trials. Any inconsistency was solved by a third reviewer. Manual searches were performed to identify relevant studies in the reference lists of the included studies.

### 2.2. Eligibility Criteria

The inclusion criteria were prespecified as (1) types of participants: patients diagnosed with immunity infertility using any recognized diagnostic criteria, regardless of age, gender, source of cases, duration of disease, ethnicity, or nationality; (2) types of interventions: TCM therapy of TKABC prescription based on the theory of “kidney deficiency and blood stasis” clearly stated in the trial group either alone or in combination with conventional treatments; no restriction was imposed on the prescription name, administration mode, dosage, and course of treatment; (3) types of comparator(s)/control: patients treated with conventional (the same conventional regimen as intervention group in the same original study), placebo, or no treatment; (4) types of outcome measures: the total effective rate for immune infertility, pregnancy rate, negative conversion rate of antibodies, and adverse effects; and (5) types of study: RCT. The exclusion criteria included (1) non-RCTs, reviews, animal-based research, conference proceedings, and literature review; (2) unclear diagnostic criteria and outcome measurements; (3) unable to get original data; (4) duplicated publications; and (5) other TCM treatments involving acupuncture and massage.

### 2.3. Outcome Measurements

Primary outcomes included the total effective rate and pregnancy rate. The secondary outcomes were defined as the negative conversion rates of antibodies (AsAb, AEmAb, AoAb, AhCGAb, and ACAb) and incidence of adverse effects.

### 2.4. Data Extraction

Two reviewers (YLB and HZW) independently screened the titles and abstracts of eligible studies and then reviewed the full text following the prespecified eligibility criteria. They independently extracted the following information by a predesigned and standardized data extraction form: first author, year of publication, sample size, gender and age, course of the disease, TCM pattern differentiation, TCM treatment interventions and control groups, treatment duration, and primary and secondary outcome measurements. Any conflict was resolved by a third author (YHC). All data were cross-checked and transferred to RevMan software (V.5.3).

### 2.5. Quality Assessment

Two reviewers (YLB and LLH) independently used the Cochrane Handbook for Systematic Reviews of Interventions to evaluate the risk of bias for the included studies in the following seven domains: random sequence generation, allocation concealment, blinding of participants and personnel, blinding of outcome assessors, incomplete outcome data, selective reporting, and other sources of bias. Each domain was assessed and graded as “low risk,” “unclear,” and “high risk.” Any disagreement was referred to a third investigator (YHC).

### 2.6. Statistical Analysis

The quantitative synthesis was performed using the Review Manager 5.3 software (The Cochrane Collaboration, NCC, CPH, Denmark). Relative risk (RR) with 95% confidence intervals (CIs) was used for binary variables, while the standard mean differences (SMD) with 95% CIs was applied for continuous variables. The chi-square statistic and *I*^2^ statistic were employed to investigate statistical heterogeneity. The fixed-effects model was used for a low heterogeneity (*I*^2^ < 50%), and the random-effects model was applied if heterogeneity was moderate (50% < *I*^2^ < 75%). Subgroup analyses were carried out to identify the potential source of high heterogeneity. Funnel plots were used to evaluate the potential reporting bias when more than ten eligible studies were included. Sensitivity analysis was conducted to assess the robustness of the pooled effects of the included studies.

## 3. Result

### 3.1. Results of Literature Search

**Figure 1:**
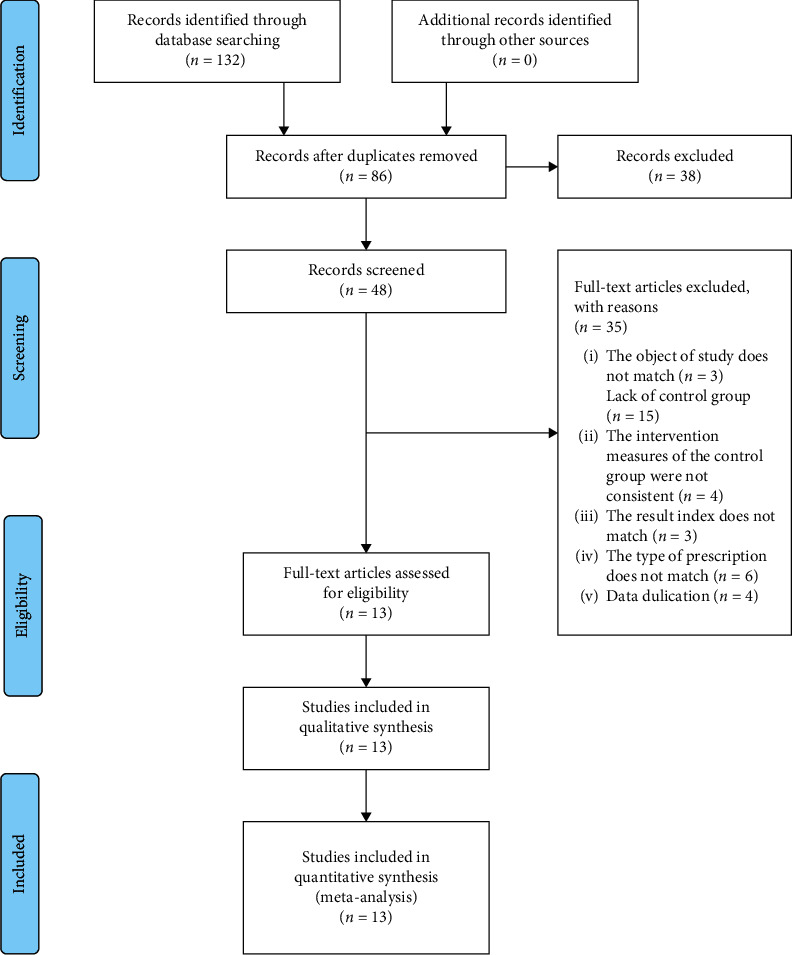
The PRISMA flowchart of the selection process.

Initially, potential 132 relevant studies were identified based on the search strategy. After excluding duplicate studies, the abstract and title of 86 studies were reviewed. Then, 48 articles were evaluated by full text, and 35 trials were excluded for the following reasons: three non-TKABC studies, 15 articles lack of control group, four studies without consistent intervention measures, three articles lack of eligible outcome measurements, six articles without the eligible type of prescription, and four articles with duplicate publication. Eventually, 13 studies were included for meta-analysis [6–18]. The flowchart of the selection process is shown in Figure 1.

### 3.2. Basic Characteristics of the Included Studies

Table 1 summarizes the basic characteristics of the included 13 trials. All the studies were conducted in China. A total of 1298 patients with immunity infertility were included [6–18], 730 in the trial group and 568 in the control group. The diagnosis of immunity infertility was clearly identified in all studies. Twelve studies were treated with herbal decoction [6–15, 17, 18], and one study was cured with Chinese patent medicine [16]. Patients in the control group were treated with Western medicine in all studies. For the outcome measurements, 12 trials presented the total effective rate [6, 8–18], 12 trials reported pregnancy rates [7–18], five trials mentioned AsAb [8, 11, 13, 14, 16], three trials presented AEmAb [12–14], one trial evaluated AoAb [13], one trial mentioned AhCGAb [13], two trials stated ACAb [7–14], and two trials reported adverse effects [11, 13]. The composition of TCM TKABC prescription in the included studies is shown in Supplementary Table 1.

### 3.3. Risk of Bias Assessment

**Figure 2:**
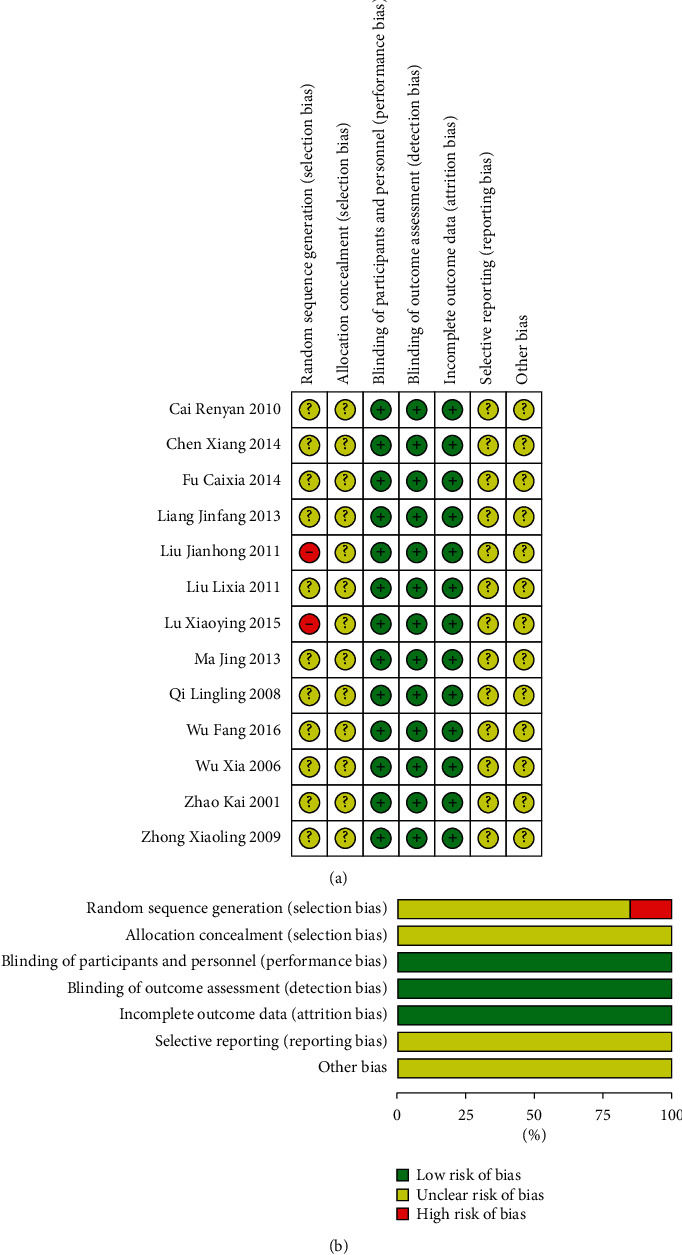
Summary of the risk of bias. The risk of bias assessment revealed that the RCTs were of poor methodological quality.

Eleven studies of the 13 studies were classified as unclear risk because they just mentioned “random” and did not describe the methods for generating method [6, 8, 9, 11–18], and two studies were considered as high risk [7, 10]. None of the studies reported the process of allocation concealment and blinding. Thus, they were rated as high risk. All the studies had complete data; hence, the attrition bias was assessed as low risk. Reporting bias and other biases were classified as unclear due to insufficient information to evaluate the risk. In summary, the quality of included RCTs was poor (Figure 2).

### 3.4. Total Effective Rate

**Figure 3:**
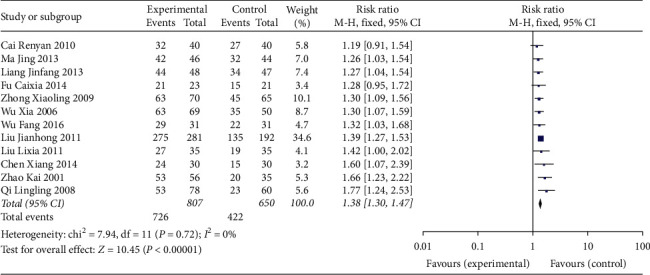
Forest plot for total effective rate between the experimental and control groups.

Twelve studies reported the total effective rate of TCM TKABC therapy in patients with immune infertility [6, 83.4; total effective rate: 18]. The pooled data of meta-analysis showed that the experimental group had a significantly higher total effective rate than that of the control group (RR: 1.38; 95% CI: 1.30, 1.47; and *I*^2^ = 0%) (Figure 3).

### 3.5. Pregnancy Rate

**Figure 4:**
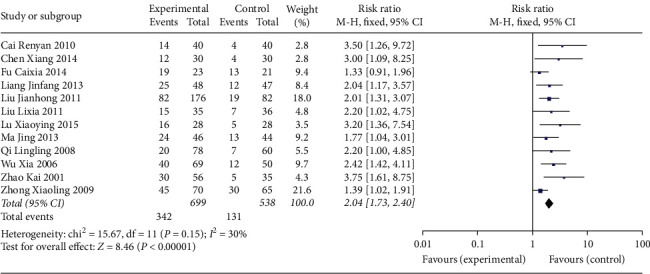
Forest plot for pregnancy rate between the experimental and control groups.

Twelve studies reported pregnancy rate [7–18]. The pooled effect of meta-analysis demonstrated that the pregnancy rate in the experimental group was significantly higher than that of the control group (RR: 2.04; 95% CI: 1.73, 2.40; and *I*^2^ = 30%) (Figure 4).

### 3.6. Negative Conversion Rate of Serum Antibody

**Figure 5:**
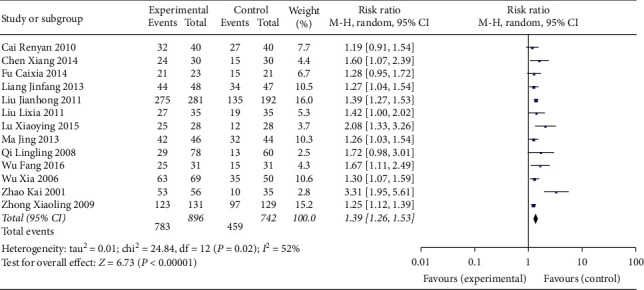
Forest plot for negative conversion rate of serum antibody between the experimental and control groups.

**Figure 6:**
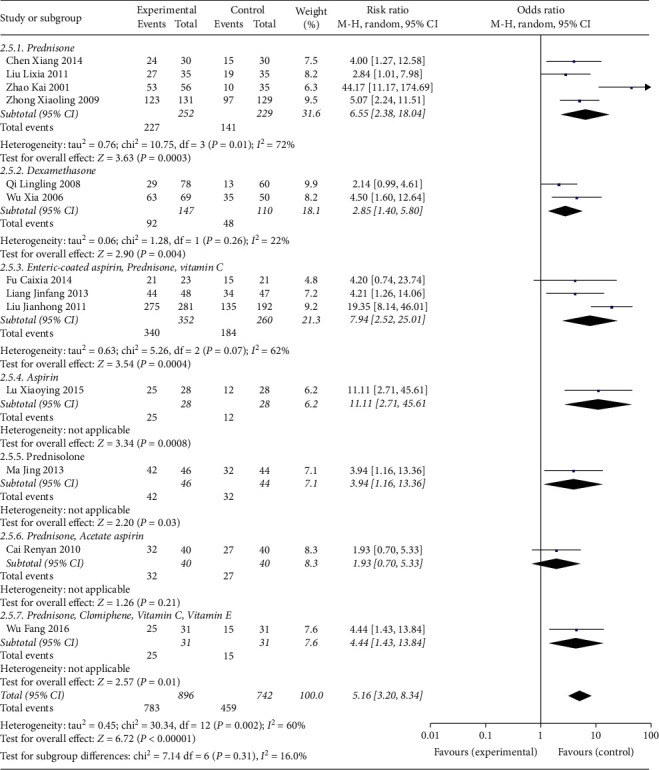
Subgroup analysis for the negative conversion rate of different comparators between the experimental and control groups.

**Figure 7:**
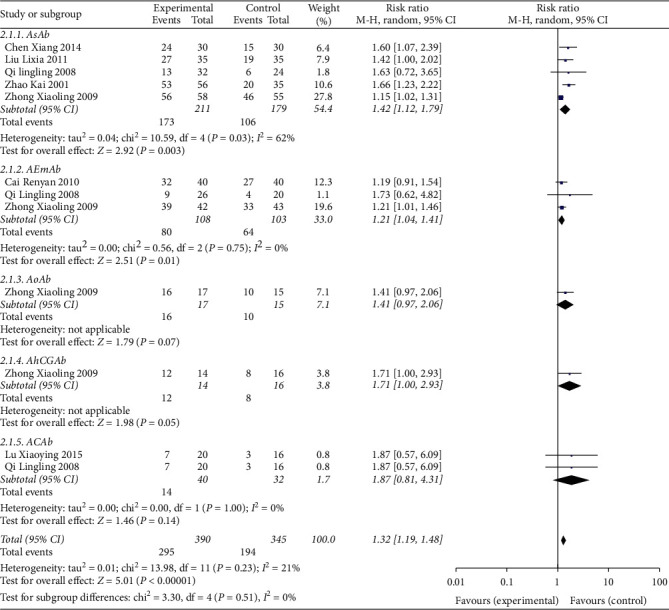
Subgroup analysis for the negative conversion rate of various serum antibody between the experimental and control groups.

All studies reported the negative conversion rate of serum antibodies. The pooled data of meta-analysis demonstrated that the negative conversion rates of serum antibodies were significantly improved in the experimental group (RR: 1.39; 95% CI: 1.26, 1.53; and *I*^2^ = 52%) (Figure 5). Subgroup analyses were performed on different comparators, as the control groups in four trials were treated with prednisone, three trials were intervened with the combination of enteric-coated aspirin, prednisone, and vitamin C, and two trials received dexamethasone therapy. The pooled data of meta-analysis revealed that the negative conversion rates of serum antibodies were significantly ameliorated in the experimental groups when compared with prednisone (RR: 6.55; 95% CI: 2.38, 18.04; and *I*^2^ = 72%) and enteric-coated aspirin, prednisone, and vitamin C (RR: 7.94; 95% CI: 2.52, 25.01; and *I*^2^ = 62%). No significant difference was evident upon comparison with the dexamethasone intervention (RR: 2.85; 95% CI: 1.40, 5.80; and *I*^2^ = 22%). The results of subgroup analyses are summarized in Figure 6. Further, subgroup analyses were carried out for serum antibodies. AsAb level was assessed in five trials, AEmAb level was measured in three trials, and ACAb level was evaluated in two trials. The pooled data of meta-analysis demonstrated that compared with the control groups, the negative conversion rates of AsAb (RR: 1.42; 95% CI: 1.12, 1.79; and *I*^2^ = 62%), AEmAb rates (RR:1.21; 95% CI: 1.04,1.41; and *I*^2^ = 0%), and AhCGAb were significantly higher in the experimental groups. No significant difference in the negative conversion rate of AoAb and ACAb (RR: 1.87; 95% CI: 0.81, 4.31; and *I*^2^ = 0%) was revealed. The results of subgroup analyses are summarized in Figure 7.

### 3.7. Adverse Effects

**Figure 8:**
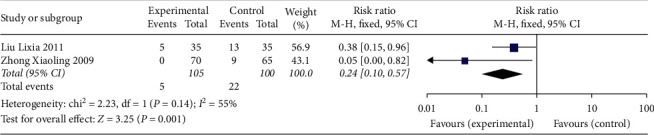
Forest plot for adverse effects between the experimental and control groups.

Two trials reported adverse effects [11, 13], including weight gain, indigestion, nausea, abdominal distension, mood changes, acne, full moon face, and flushing. The pooled effect of meta-analysis showed that compared with the control group, the adverse effects of the experimental group were significantly lower (RR: 0.24; 95% CI: 1.73, 2.40; and *I*^2^ = 55%) (Figure 8).

### 3.8. Publication Bias

**Figure 9:**
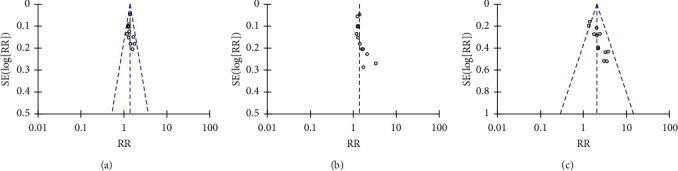
Funnel plots for publication bias. (a) Total effective rate. (b) Negative conversion rate of serum antibody. (c) Pregnancy rate.

Funnel plots were used to measure the publication bias. The total effective rate, antibody negative conversion rate, and pregnancy rate were in asymmetric distribution, indicating that publication bias might exist (Figure 9).

### 3.9. Sensitivity Analysis

Sensitivity analysis was performed for the total effective rate, the negative conversion rate of antibody, and pregnancy rate. The effect remained unchanged, indicating the robustness of the pooled results.

## 4. Discussion

According to TCM theory, the etiology and pathogenesis of immune infertility are dominated by kidney deficiency and blood stasis. The kidney is considered as “the origin of congenital constitution.” It is the origin of yin-yang, the source of life, stores the essence, and acts as the primary material foundation for the growth, development, and reproduction of human beings. Long-term kidney deficiency may cause blood stasis, and blood stasis may aggravate kidney deficiency [19–22]. Therefore, the fundamental therapeutic principles for immune infertility treatment are to tonify kidney, activate blood circulation, remove blood stasis, and dredge collaterals. Correlation analyses revealed that kidney-tonifying and blood circulation-activating prescriptions and herbs are commonly used to treat immune infertility and can regulate the reproductive axis in a bidirectional manner, the immune function, and serum antibodies [23–25]. In immune infertility, AsAb is a complex pathological product. Sperm is an antigen that causes the body to produce AsAb when the immunity system is exposed to it. AsAb reduces sperm motility, prevents sperm from undergoing capacitation and acrosome reactions, and impacts sperm-oocyte recognition and fusion [26, 27]. In this meta-analysis, we found that TKABC therapy based on the theory of kidney deficiency and blood stasis could significantly improve the total effective rate, the negative conversion rate of AsAb, AEmAb, and AhCGAb, and pregnancy rate with fewer adverse effects.

Although the effectiveness and safety of TKABC on immune infertility were evaluated using a meta-analysis, this study has several limitations. (1) The number of included studies and sample size of the studies were small. (2) Some RCTs had low methodological quality and may result in overestimation of the therapeutic effect. (3) Although we searched the studies without language limitations, all the publication regions were in China. (4) The herbal components of TKABC therapy were different among studies, which might cause bias. (5) The criteria for the efficacy and duration of treatment in each study were inconsistent. (6) Studies with negative results may have been published with a lower frequency and cause publication bias.

## 5. Conclusion

In summary, this study shows that TCM therapy of TKABC based on the theory of “kidney deficiency and blood stasis” may be effective and safe for immune infertility. It might be considered as a complementary and alternative treatment to conventional therapy. However, due to limited data and the low quality of methodology of the included studies, more well-designed and high-quality multicenter RCTs with a larger sample size need to be performed to confirm these results.

## Figures and Tables

**Table 1:** Basic characteristics of the included study.

Author(s)	Sample size Expt./Ctrl.	Average age (*y*) Expt./Ctrl.	Gender	Diagnostics	Intervention measures		Duration treatment	Outcome measures
					Expt.	Ctrl.		
Wu [6]	31/31	32.6/31.4	Female	A + B + C	Bushen Huoxue decoction + WM	Prednisone + clomiphene + vitamins C, E	3m ∗ 2	①
Lu and Gong [7]	28/28	26.34/27.12	Female	A + C	Xiaokang II decoction	Aspirin	2m ∗ 2	⑥⑦
Chen and Xu [8]	30/30	27.03/26.67	Female	A + B + C	Bushenyikang decoction	Prednisone	1m ∗ 3	①②⑦
Ma and Zhang [9]	46/44	31.2/31.4	Female	A + B + C	Yikang Zhuyun decoction	Prednisolone	14d ∗ 3	①⑦
Liu et al. [10]	176/82	33.5/33.5	Female	A + B + C	Bushen Huoxue decoction + WM	Vitamin C + aspirin + prednisone	21d ∗ 2	①⑦
Liu [11]	35/35	29.33/29.25	Female	A + B + C	Bushen Huoxue decoction + WM	Prednisone	3m ∗ 3	①②⑦⑧
Cai et al. [12]	40/40	28.76/30.72	Female	A + B + C	Huoxue Xiaokang decoction	Prednisone + acetate aspirin	45d ∗ 2	①③⑦
Zhong et al. [13]	70/65	27.8/27.8	Female	A + B + C	Bushen Huoxue decoction	Prednisone	1m ∗ 3	①②③④⑤⑦⑧
Qi et al. [14]	78/60	29.5/29.5	Female	A + B + C	Yulin Qingkang decoction	Dexamethasone	1m × 3	①②③⑥⑦
Wu [15]	69/50	28.35/28.35	Female	A + B + C	Assisting-pregnancy decoction	Dexamethasone	2m × 1	①⑦
Zhao [16]	56/35	28.35/28.35	Female	A + B + C	Anti-immunity I tablet	Prednisone	2m ∗ 3	①②⑦⑧
Fu [17]	23/21	28.1/28.1	Female	A + B + C	Bushen Huoxue Xiaokang decoction + WM	Enteric-coated aspirin + prednisone + vitamin C	14d ∗ 1	①⑦
Liang and Yuan [18]	48/47	—	Female	A + B + C	Bushen Huoxue Xiaokang decoction + WM	Enteric-coated aspirin + prednisone + vitamin C	14d ∗ 2	①⑦

Expt.: experimental group; Ctrl.: control group; A: diagnostic criteria for infertility: unable to conceive after one year or longer of unprotected sex; B: ruling out infertility due to other factors, such as tubal obstruction, ovulation disorders, and endometriosis; C: positive for at least one of the following serum antibody tests: AsAb, AEmAb, ACAb, AoAb, AZPAb, and AhCGAb. ① Total effective rate; ② serum AsAb negative conversion rate; ③ serum AEmAb negative conversion rate; ④ serum AoAb negative conversion rate; ⑤ serum AhCGAb negative conversion rate; ⑥ ACAb negative conversion rate; ⑦ pregnancy rate; and ⑧ adverse effects.

## Supplementary Materials

Supplementary Materials
*Supplementary Table 1*. Composition of TCM prescription in the included studies.
