# Supplementary material for: Efficacy and Safety of Traditional Chinese Medicine in the Treatment of Immune Infertility Based on the Theory of “Kidney Deficiency and Blood Stasis”: A Systematic Review and Meta-Analysis
Source: Evid Based Complement Alternat Med. 2021 May 15;2021:9947348. doi: 10.1155/2021/9947348 (PMC8149227; doi:10.1155/2021/9947348)
Supplement: Supplementary Materials — Supplementary Table 1. Composition of TCM prescription in the included studies. [file 9947348.f1.docx]

Supplement Table 1: Composition of TCM prescription in the included studies

| Author(s) | Experimental Group | Composition |
| --- | --- | --- |
| Wu et al. [6] | Bushen Huoxue  Decoction | Rehmanniae Radix (Shudihuang) 20g, Angelicae Sinensis Radix (Danggui) 10g, Atractylodis Macrocephalae Rhizoma (Baizhu) 10g, Salviae Miltiorrhizae Radix et Rhizoma (Danshen) 10g, Chuanxiong Rhizoma (Chuanxiong) 10g, Dipsaci Radix (Xuduan) 10g, Morindae Officinalis Radix (Bajitian) 10g, Semen Cuscutae (Tusizi) 10g |
| Lu et al. [7] | Xiaokang II  Decoction | Taxilli Herba (Sangjisheng) 15g, Dipsaci Radix (Xuduan) 15g, Semen Cuscutae (Tusizi) 30g, Paeoniae Radix Alba (Baishao) 15g, Scutellariae Radix (Huangqin) 10g, Cistanches Herba (Roucongrong) 10g, Cibotii Rhizoma (Gouji) 10g, Salviae Miltiorrhizae Radix et Rhizoma (Danshen) 15g, Paeoniae Radix Rubra (Chishao) 10g |
| Chen et al. [8] | Bushen Yikang Decoction | Epimedii Folium (Yinyanghuo) 15g, Cervi Cornua Degelatinatum (Lujiaoshuang) 15g, Corni Fructus (Shanzhuyu) 15g, Dioscoreae Rhizoma (Shanyao) 30g, Astragali Radix (Huangqi) 15g, Angelicae Sinensis Radix (Danggui) 10g, Moutan Cortex (Mudanpi) 10g, Paeoniae Radix Alba (Baishao) 10g, Poria (Fuling) 10g |
| Ma et al. [9] | Yikang Zhuyun Decoction | Rehmanniae Radix (Shengdihuang) 12g, Corni Fructus (Shanzhuyu) 15g, Dioscoreae Rhizoma (Shanyao) 12g, Moutan Cortex (Mudanpi) 9g, Alismatis Rhizoma (Zexie) 9g, Poria (Fuling) 9g, Ligustri Lucidi Fructus (Nüzhenzi) 15g, Herba Echiptae (Hanliancao) 15g, Salviae Miltiorrhizae Radix et Rhizoma (Danshen) 9g, Angelicae Sinensis Radix (Danggui) 9g, Paeoniae Radix Rubra (Chishao) 9g, Paeoniae Radix Alba (Baishao) 15g, Astragali Radix (Huangqi) 15g, Atractylodis Macrocephalae Rhizoma (Baizhu) 15g, Saposhnikoviae Radix (Fangfeng) 9g, Radix Boehmeriae (Zhumagen) 30g, Scutellaria Barbata Don (Banzhilian) 15g, Taraxaci Herba (Pugongying) 15g |
| Liu et al. [10] | Bushen Huoxue  Decoction | Semen Cuscutae (Tusizi) 15g, Herba Echiptae (Hanliancao) 10g, Ligustri Lucidi Fructus (Nüzhenzi) 10g, Chrysanthemi Flos (Juhua) 10g, Lonicerae Japonicae Flos (Jinyinhua) 10g, Paeoniae Radix Rubra (Chishao) 10g, Paeoniae Radix Alba (Baishao) 10g, Angelicae Sinensis Radix (Danggui) 10g, Astragali Radix (Huangqi) 15g, Herba Verbenae Officinalis (Mabiancao) 15g, Saposhnikoviae Radix (Fangfeng) 6g, Chuanxiong Rhizoma (Chuanxiong) 10g, Salviae Miltiorrhizae Radix et Rhizoma (Danshen) 10g, etc. |
| Liu et al. [11] | Bushen Huoxue  Decoction | Corni Fructus (Shanzhuyu) 15g, Angelicae Sinensis Radix (Danggui) 15g, Rehmanniae Radix (Shengdihuang) 15g, Lycii Fructus (Gouqizi) 15g, Spatholobi Caulis (Jixueteng) 15g, Poria (Fuling) 15g, Persicae Semen (Taoren) 9g, Carthami Flos (Honghua) 5g, Cyperi Rhizoma (Xiangfu) 6g, Moutan Cortex (Mudanpi) 9g, Paeoniae Radix Rubra (Chishao) 9g |
| Cai et al. [12] | Huoxue Xiaokang Decoction | Rehmanniae Radix (Shengdihuang) 12g, Angelicae Sinensis Radix (Danggui) 12g, Chuanxiong Rhizoma (Chuanxiong) 10g, Paeoniae Radix Rubra (Chishao) 15g, Persicae Semen (Taoren) 10g, Carthami Flos (Honghua) 10g, Salviae Miltiorrhizae Radix et Rhizoma (Danshen) 30g, Spatholobi Caulis (Jixueteng) 30g, Bupleuri Radix (Chaihu) 6g, Aurantii Fructus (Zhiqiao) 10g, Herba Verbenae Officinalis (Mabiancao) 30g, Semen Cuscutae (Tusizi) 30g, Glycyrrhizae Radix et Rhizoma (Gancao) 6g |
| Zhong et al. [13] | Bushen Huoxue  Decoction | Rehmanniae Radix (Shudihuang) 30g, Rehmanniae Radix (Shengdihuang) 30g, Astragali Radix (Huangqi) 15g, Paeoniae Radix Rubra (Chishao) 15g, Angelicae Sinensis Radix (Danggui) 10g, Persicae Semen (Taoren) 10g, Semen Cuscutae (Tusizi) 15g, Epimedii Folium (Yinyanghuo) 15g, Salviae Miltiorrhizae Radix et Rhizoma (Danshen) 15g, Lycii Fructus (Gouqizi) 10g, Cyperi Rhizoma (Xiangfu) 10g, Glycyrrhizae Radix et Rhizoma (Gancao) 10g |
| Qi et al. [14] | Yulin Qingkang Decoction | Semen Cuscutae (Tusizi) 10g, Eucommiae Cortex (Duzhong) 15g, Paeoniae Radix Rubra (Chishao) 10g, Chuanxiong Rhizoma (Chuanxiong) 10g, Angelicae Sinensis Radix (Danggui) 10g, Ginseng Radix et Rhizoma (Renshen) 10g, Atractylodis Macrocephalae Rhizoma (Baizhu) 10g, Glycyrrhizae Radix et Rhizoma (Gancao) 6g |
| Wu et al. [15] | Assisting-pregnancy Decoction | Astragali Radix (Huangqi) 15g, Salviae Miltiorrhizae Radix et Rhizoma (Danshen) 15g, Angelicae Sinensis Radix (Danggui) 10g, Rehmanniae Radix (Shudihuang) 10g, Paeoniae Radix Rubra (Chishao) 10g, Persicae Semen (Taoren) 10g, Carthami Flos (Honghua) 10g, Cyperi Rhizoma (Xiangfu) 10g, Alpiniae Oxyphyllae Fructus (Yizhi) 10g, Semen Cuscutae (Tusizi) 10g, Lycii Fructus (Gouqizi) 10g, Epimedii Folium (Yinyanghuo) 10g, Radix Boehmeriae (Zhumagen) 20g, Phellodendri Chinensis Cortex (Huangbo) 9g, Glycyrrhizae Radix et Rhizoma (Gancao) 3g |
| Zhao et al. [16] | Anti-immunity Ⅰ  Tablet | Rehmanniae Radix (Shengdihuang) 10g, Lycii Fructus (Gouqizi) 10g, Morindae Officinalis Radix (Bajitian) 10g, Salviae Miltiorrhizae Radix et Rhizoma (Danshen) 10g, Drynariae Rhizoma (Gusuibu) 15g, Moutan Cortex (Mudanpi) 15g, Curcumae Rhizoma (Ezhu) 15g, Radix Cynanchi Paniculati (Xuzhangqing) 15g, Semen Cuscutae (Tusizi) 20g, Fritillariae Thunbergii Bulbus (Zhebeimu) 20g, Ostreae Concha (Muli) 30g, Glycyrrhizae Radix et Rhizoma (Gancao) 5g |
| Fu et al. [17] | Bushen Huoxue Xiaokang Decoction | Rehmanniae Radix (Shengdihuang) 12g, Dioscoreae Rhizoma (Shanyao) 10g, Ligustri Lucidi Fructus (Nüzhenzi) 12g, Corni Fructus (Shanzhuyu) 10g, Herba Echiptae (Hanliancao) 15g, Glycyrrhizae Radix et Rhizoma (Gancao) 6g, Moutan Cortex (Mudanpi) 10g, Semen Cuscutae (Tusizi) 15g |
| Liang et al. [18] | Bushen Huoxue Xiaokang Decoction | Rehmanniae Radix (Shudihuang) 30g, Rehmanniae Radix (Shengdihuang) 30g, Dioscoreae Rhizoma (Shanyao) 15g, Corni Fructus (Shanzhuyu) 15g, Poria (Fuling) 12g, Moutan Cortex (Mudanpi) 12g, Alismatis Rhizoma (Zexie) 12g, Salviae Miltiorrhizae Radix et Rhizoma (Danshen) 30g, Persicae Semen (Taoren) 10g, Paeoniae Radix Rubra (Chishao) 15g, Angelicae Sinensis Radix (Danggui) 12g, Astragali Radix (Huangqi) 15g, Lycii Fructus (Gouqizi) 30g, Semen Cuscutae (Tusizi) 20g, Cyperi Rhizoma (Xiangfu) 12g, Cervi Cornua Degelatinatum (Lujiaoshuang) 15g, Alpiniae Oxyphyllae Fructus (Yizhi) 15g |
